# THE PERCEIVED LIFE EXPERIENCES OF AFRICAN METHODIST EPISCOPAL (AME) MEMBERS

**DOI:** 10.1093/geroni/igae098.4318

**Published:** 2024-12-31

**Authors:** Nia Lane-Nelson

**Affiliations:** Hallmark University, San Antonio, Texas, United States

## Abstract

The African American (AA) church has played an essential role in the AA community. Using the Life Course Theory (LCT) as a sensitizing framework, this qualitative descriptive study described how AA members of one AME church perceived their life experiences as influencing their current health status. LCT key concepts (pathways or trajectories, early life experiences, critical or sensitive periods, cumulative impacts, and risk and protective factors) concentrate on the socioeconomic and environmental contexts within the life of an individual. Individual interview questions were developed based on the LCT key concepts. Seventeen in-depth interviews were conducted. Qualitative content analysis was used to identify categories and subcategories. Within the key concept of early life experiences 3 categories (Economic hardships, Personal hardships, and Protective influences in early childhood) were identified. Within the category of Economic hardships 3 subcategories were identified; not having food, living was tight, and neglect in early childhood due to economic hardships. Within Personal hardships the subcategories were parental responsibilities in early childhood, separation in family, family trauma, and experiences with racism at an early age. Within Protective influences in early childhood the subcategories were receiving love and support, feeling safe and protected, learning from parents and others, dealing with racism, and active in childhood activities. Through using the LCT, participants were able to highlight the social, economic, and environmental issues experienced throughout their lives and the impact on their health. Participants shared narratives spanning from early childhood to the present, with a common stressor being experiences with racial discrimination.

